# Marine Algae Hydrogels as Emerging Biomaterials for Medicine

**DOI:** 10.3390/gels12030228

**Published:** 2026-03-11

**Authors:** Leonel Pereira, Ana Valado

**Affiliations:** 1 Centre for Functional Ecology (CFE), Marine Resources, Conservation and Technology, Marine Algae Lab, Department of Life Sciences, University of Coimbra, 3000-456 Coimbra, Portugal; 2 Associate Laboratory TERRA, Centre for Functional Ecology—Science for People & the Planet (CFE), University of Coimbra, Campus at Figueira da Foz, Quinta das Olaias, 3080-183 Figueira da Foz, Portugal; 3 Polytechnic University of Coimbra, Rua da Misericórdia, Lagar dos Cortiços, S. Martinho do Bispo, 3045-093 Coimbra, Portugal; valado@estesc.ipc.pt; 4 H&TRC—Health & Technology Research Center, Coimbra Health School, Polytechnic University of Coimbra, Rua 5 de Outubro, 3045-043 Coimbra, Portugal; 5 Research Center for Natural Resources, Environment and Society (CERNAS), Polytechnic University of Coimbra, Bencanta, 3045-601 Coimbra, Portugal; 6 MARE—Marine and Environmental Sciences Centre/ARNET—Aquatic Research Network, University of Coimbra, 3000-456 Coimbra, Portugal

**Keywords:** hydrogels, phycocolloids, tissue engineering, drug delivery, regenerative medicine, biomaterials, 3D bioprinting

## Abstract

Marine algae, microalgae, and Cyanophyceae emerge as sustainable and versatile sources of biomacromolecules for the fabrication of hydrogels with broad biomedical potential. Their phycocolloids, such as alginate, agar, carrageenan, ulvan, and extracellular polysaccharides (EPS), exhibit intrinsic biocompatibility, tunable gelation behavior, and bioactive sulfated structures that support cell viability, tissue regeneration, and therapeutic delivery. This review provides a comprehensive overview of hydrogel fabrication strategies, including physical, chemical, and hybrid crosslinking approaches, and highlights recent advances in composite systems incorporating proteins, glycosaminoglycans, and functional nanomaterials. Applications in skin repair, cartilage and bone regeneration, neural and cardiovascular engineering, and controlled drug delivery are examined, alongside the expanding role of marine-derived hydrogels as bioinks for 3D and 4D bioprinting. Despite their promise, challenges remain related to extract variability, purification complexity, mechanical limitations, and the need for standardized characterization. Future perspectives emphasize genetic engineering of algae and cyanobacteria, development of multifunctional hybrid hydrogels, sustainable large-scale production, and pathways toward clinical translation. Together, these insights position marine-derived hydrogels as next-generation biomaterials with significant potential for regenerative medicine and therapeutic innovation.

## 1. Introduction

Hydrogels have become indispensable in tissue engineering and biomedical therapeutics due to their ability to replicate the hydrated, viscoelastic nature of native tissues. Their high-water content, compatibility, and adjustable mechanical properties make them ideal scaffolds for cell encapsulation, proliferation, and differentiation. Among the diverse hydrogel sources, phycocolloids extracted from marine algae, microalgae, and Cyanophyceae have gained significant attention as sustainable, renewable, and functionally rich biomaterials [1].

Marine ecosystems provide an abundant reservoir of structurally unique polysaccharides with intrinsic bioactivities, including antioxidant, anti-inflammatory, antiviral, and immunomodulatory properties [2]. These natural polymers, such as alginate, agar, carrageenan, fucoidan, ulvan, and cyanobacterial exopolysaccharides, offer versatile chemical functionalities that enable precise control over gelation, degradation, and mechanical behavior [3]. Their ecological sustainability and low environmental footprint further enhance their appeal for biomedical applications [4].

This review synthesizes the latest advances in the design, fabrication, and application of phycocolloid-based hydrogels, emphasizing their role in tissue engineering and therapeutic delivery. It also highlights emerging trends, challenges, and future directions in this rapidly evolving field.

## 2. Phycocolloids from Marine Algae and Microalgae

### 2.1. Alginate

**Figure 1:**
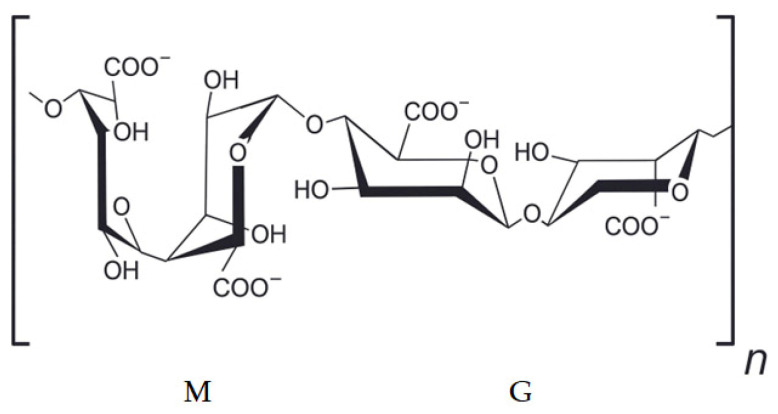
Structure of Alginic Acid.

Alginate is one of the most extensively studied and widely applied marine-derived polysaccharides in biomedical hydrogel research. Extracted predominantly from brown macroalgae such as *Laminaria*, *Ascophyllum*, and *Macrocystis* (Phaeophyceae) species, alginate is composed of alginic acid, linear copolymers of β-D-mannuronic acid (M) and α-L-guluronic acid (G) residues arranged in homopolymeric (M-blocks, G-blocks) (Figure 1) or heteropolymeric (MG-blocks) sequences [5] (Figure 1). The relative proportion and distribution of these blocks strongly influence the physicochemical behavior of alginate, particularly its gelation capacity, mechanical strength, and degradation profile. This structural versatility, combined with its natural abundance and low extraction cost, has positioned alginate as a cornerstone material in the development of hydrogels for tissue engineering and biomedical therapeutics.

One of alginate’s most attractive features is its ability to undergo rapid ionic crosslinking in the presence of divalent cations such as Ca^2+^, Ba^2+^, or Sr^2+^. This mild gelation process occurs under physiological conditions and does not require harsh chemicals, high temperatures, or organic solvents, making it highly suitable for encapsulating living cells, bioactive molecules, and sensitive therapeutic agents [6]. The resulting hydrogels exhibit high water content, configurable viscoelasticity, and a porous network that supports nutrient diffusion and waste removal, properties essential for maintaining cell viability and promoting tissue regeneration. By adjusting parameters such as alginate concentration, molecular weight, G/M ratio, and crosslinker type, researchers can finely modulate the mechanical stiffness, degradation rate, and structural integrity of the hydrogel to match the requirements of specific tissues [7].

Despite its many advantages, native alginate lacks intrinsic cell-adhesive motifs, which can limit cell attachment and spreading. To overcome this limitation, extensive efforts have been devoted to chemical modification strategies that enhance alginate’s biofunctionality [8]. Methacrylation, for example, enables photo-crosslinking and the fabrication of hydrogels with improved mechanical stability and spatial patterning capabilities [9]. Oxidation of alginates introduces aldehyde groups that increase degradability and facilitate covalent bonding with proteins or peptides. Conjugation with cell-adhesive ligands such as cyclic arginylglycylaspartic acid peptide (c-RGD) peptides significantly improves cellular interactions, promoting adhesion, proliferation, and differentiation. These modifications have expanded alginate’s applicability across a wide range of biomedical contexts, including cartilage and bone regeneration, wound healing, and controlled drug delivery [10].

Alginate has also become a leading material in the field of 3D bioprinting due to its shear-thinning behavior, rapid gelation, and cytocompatibility. It can be extruded into complex architectures that maintain structural fidelity while supporting encapsulated cells [11]. Hybrid bioinks combining alginate with gelatin, collagen, nanocellulose, or other biopolymers further enhance printability, mechanical performance, and biological activity. Such composite systems are increasingly used to fabricate patient-specific scaffolds, vascularized constructs, and organ-on-chip platforms [12].

Overall, alginate’s unique combination of safety, tunability, and ease of processing continues to drive innovation in hydrogel-based tissue engineering and therapeutic delivery. Ongoing research into its chemical modification, crosslinking strategies, and integration with emerging technologies such as 3D bioprinting and bioactive nanomaterials is expected to further expand its potential in next-generation biomedical applications [8].

### 2.2. Agar and Agarose

**Figure 2:**
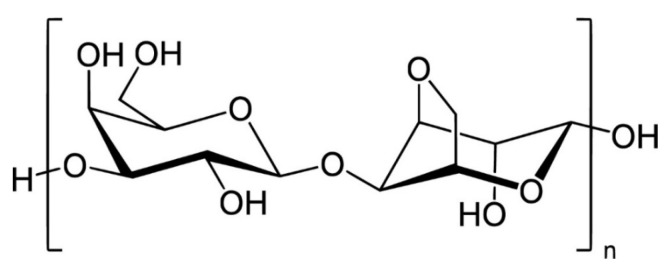
Agarose.

Agar and agarose (Figure 2) are polysaccharides extracted primarily from red algae and are widely recognized for their ability to form thermo-reversible hydrogels. These materials exhibit high mechanical strength, structural stability, and low toxicity, making them particularly attractive for biomedical and biotechnological applications. Their gelation behavior, driven by temperature-dependent molecular self-assembly, enables the formation of stable yet easily processable matrices suitable for a variety of engineering and biological contexts [13].

Due to these characteristics, agar- and agarose-based hydrogels have been extensively used as tissue scaffolds, where their porosity support cell adhesion, proliferation, and nutrient diffusion. In drug delivery, their controlled gelation and diffusion properties allow for the sustained release of therapeutic agents [14]. Agarose also plays a central role in microfluidic systems, where its mechanical robustness and ease of molding facilitate the fabrication of channels and compartments for analytical and diagnostic devices [15]. Additionally, its inert nature and optical clarity make agarose a standard substrate for cell culture platforms, enabling the study of cellular behavior in three-dimensional environments [16].

### 2.3. Carrageenan

**Figure 3:**
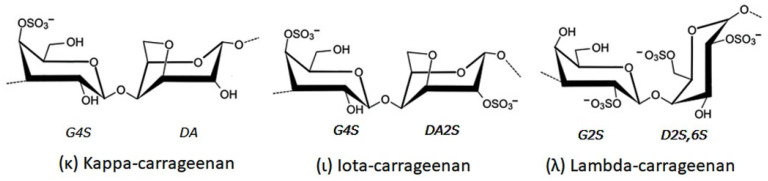
Main Carrageenan types (These units are composed of a 1,3-linked β-d-galactopyranose (G-unit) and a 1,4-linked α-d-galactopyranose (D-unit) or 3,6-anhydro-α-d galactopyranose (DA-unit), and G4S for a 1,3-linked galactose unit which are sulfated at position C4).

Carrageenans are sulfated galactans derived from red algae and are typically classified into three major types, κ-, ι-, and λ-carrageenan (Figure 3), each distinguished by its sulfate content and resulting rheological behavior. These polysaccharides are well known for their ability to form viscoelastic hydrogels, with their sulfate groups playing a central role in determining gel strength, solubility, and interaction with biological molecules. The presence of these charged groups not only influences the physical properties of carrageenan-based materials but also imparts intrinsic bioactivity, enabling selective interactions with proteins, growth factors, and components of the extracellular matrix.

Because of these characteristics, carrageenans have gained considerable attention in biomedical engineering. They are widely used in injectable hydrogels, where their shear-thinning behavior allow minimally invasive administration and in situ gelation. In wound dressings, carrageenan-based materials provide moisture retention, mechanical protection, and potential bioactive effects that support tissue repair. Their ability to promote rapid clot formation has also led to the development of carrageenan-derived hemostatic materials. Furthermore, their network structure makes them suitable for controlled release systems, enabling the sustained delivery of therapeutic agents through diffusion- or degradation-driven mechanisms [17].

### 2.4. Ulvan

**Figure 4:**
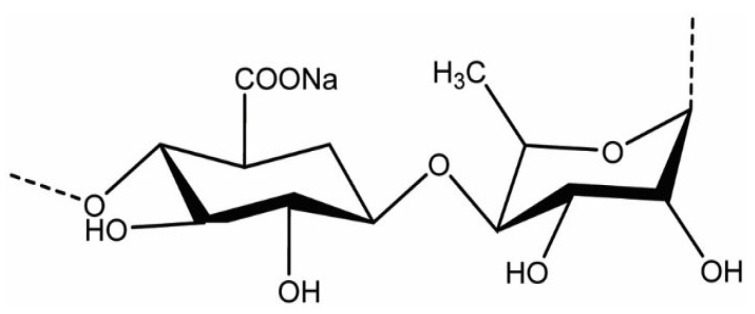
Ulvan.

Ulvan (Figure 4) is a sulfated polysaccharide extracted from green algae of the *Ulva* genus (Chlorophyta), and it has attracted considerable scientific interest due to its diverse biological activities [18]. Research highlights its antioxidant, antiviral, and immunomodulatory potential, making it a valuable candidate for biomedical and biotechnological applications. In recent years, ulvan-based hydrogels have emerged as particularly promising materials. Their ability to form stable, functional networks supports their use in skin regeneration, where they can promote healing and tissue repair. They also show potential as antimicrobial coatings, helping to inhibit microbial growth on medical devices or surfaces, and as bioactive scaffolds capable of supporting cell adhesion and proliferation in tissue engineering contexts [19].

Despite these advantages, ulvan’s structural complexity poses challenges. Its composition can vary depending on species, environmental conditions, and extraction procedures, which makes standardization difficult. As a result, advanced extraction and purification techniques are essential to obtain reproducible, well-defined ulvan fractions suitable for consistent research and industrial use [20].

### 2.5. Fucoidan and Laminarin

**Figure 5:**
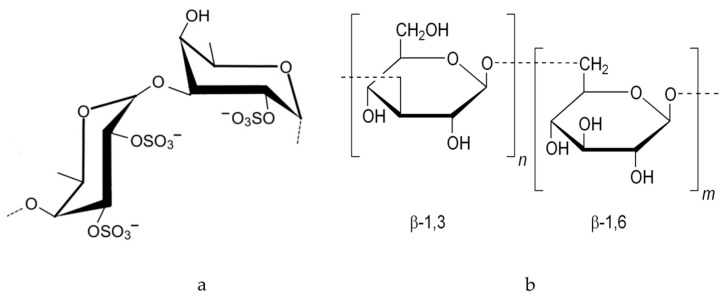
Fucoidan (**a**), Laminarin (**b**).

Fucoidan and laminarin, two bioactive polysaccharides derived from brown seaweeds (Phaeophyceae), have gained prominence in biomedical research. Fucoidan is a sulfated fucose-rich polymer known for its anti-inflammatory, antiviral, anticoagulant, and anticancer properties. Laminarin, a β-glucan composed of glucose units linked by β-1,3 and β-1,6 bonds, exhibits antioxidant, immunomodulatory, and wound-healing effects (Figure 5). Both compounds demonstrate excellent biocompatibility and low toxicity, making them suitable for applications in tissue engineering, drug delivery, and oncology. Their inclusion expands the functional diversity of marine-derived hydrogels and supports the development of multifunctional therapeutic platforms [21].

Fucoidan, rich in sulfated fucose units, exhibits potent anti-inflammatory, antiviral, and anticoagulant activities, making it a valuable candidate for wound healing, cancer therapy, and cardiovascular protection. Its ability to modulate immune responses and inhibit viral replication supports its use in topical formulations and drug delivery systems. Laminarin is known for its antioxidant, immunomodulatory, and tissue-regenerative effects. It has been explored in skin repair, immune support, and as a scaffold material in tissue engineering, particularly for its biocompatibility and ability to promote cellular proliferation. Together, these marine-derived polysaccharides expand the functional landscape of hydrogels, offering versatile platforms for therapeutic innovation and regenerative medicine [22].

Extraction and purification of marine polysaccharides play a critical role in determining their structural integrity, bioactivity, and suitability for hydrogel fabrication. Common extraction approaches include hot-water extraction, acid or alkaline treatments, and enzyme-assisted processes, each selected according to the specific cell-wall architecture of brown, red, or green algae. Purification typically involves sequential steps such as filtration, ethanol precipitation, dialysis, and chromatographic fractionation to remove proteins, pigments, salts, and low-molecular-weight impurities. As noted in the manuscript, the composition of ulvan, fucoidan, and other phycocolloids can vary significantly with species and environmental conditions, making standardized extraction protocols essential for reproducibility. Recent advances also include green extraction technologies, such as microwave-assisted, ultrasound-assisted, and supercritical fluid extraction, which improve yield while preserving functional groups relevant for hydrogel formation. Together, these methods ensure the production of well-defined, high-purity polysaccharide fractions suitable for biomedical applications [4].

## 3. Cyanobacterial Polysaccharides and Exopolysaccharides

**Figure 6:**
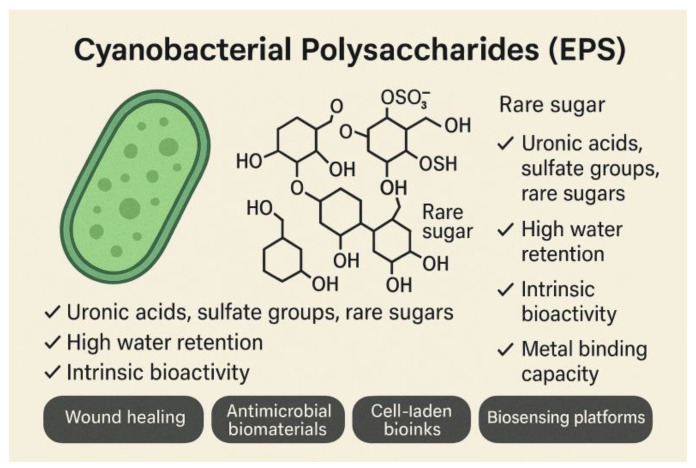
Extracellular polysaccharides (EPS) bioactivities.

Cyanophyceae are prolific producers of extracellular polysaccharides (EPS), generating a remarkably diverse set of biopolymers enriched with uronic acids, sulfate groups, and various rare sugars. This chemical complexity gives cyanobacterial EPS distinctive physicochemical and biological properties. They are known for their exceptional water-retention capacity, which supports moisture maintenance in biomedical applications, and for their intrinsic bioactivity, including antioxidant and anti-inflammatory effects (Figure 6). Another notable feature is their strong metal-binding ability, which has implications for bioremediation and environmental technologies [23].

Building on these characteristics, cyanobacterial hydrogels emerging as versatile materials across several advanced fields. In wound healing, they offer a moist, protective environment that can support tissue repair while providing bioactive cues [24]. Their antimicrobial potential makes them attractive for developing protective biomaterials that limit pathogen growth. In bio-fabrication, cyanobacterial EPS are being explored as components of cell-laden bioinks, where their rheological properties support 3D bioprinting. Additionally, their responsiveness to environmental stimuli positions them as promising candidates for biosensing platforms [25].

The expanding use of cyanobacterial polysaccharides is further propelled by advances in genetic engineering. By modifying metabolic pathways, researchers can tailor EPS composition, finetune functional groups, and enhance hydrogel performance. This ability to design customized biopolymers opens new avenues for creating next-generation materials with optimized mechanical, biological, and chemical properties [26].

## 4. Hydrogel Fabrication and Crosslinking Strategies

Hydrogel formation from marine algae, microalgae, and Cyanophyceae relies on a range of crosslinking strategies that determine the material’s mechanical properties, stability, and suitability for biomedical applications. Among these, physical crosslinking methods are particularly attractive for tissue engineering because they avoid harsh chemical reagents and preserve the bioactivity of incorporated cells, proteins, or therapeutic molecules. These approaches exploit the intrinsic physicochemical behavior of algal polysaccharides, such as alginate, agar, and carrageenan, to form three-dimensional networks under mild, biocompatible conditions. As a result, physically crosslinked hydrogels are frequently used in injectable systems, cell encapsulation, and in situ gelling formulations where cytocompatibility and structural reversibility are essential [1,4].

### 4.1. Physical Crosslinking

Physical crosslinking encompasses several non-covalent mechanisms that drive gel formation without the need for chemical initiators or crosslinkers. One of the most widely used strategies is ionic gelation, exemplified by the rapid formation of alginate hydrogels upon exposure to divalent cations such as Ca^2+^. This process produces stable yet permeable networks capable of supporting cell viability, making Ca^2+^–alginate systems a cornerstone of marine-derived biomaterials [27]. Another important mechanism is thermo-reversible gelation, characteristic of agar and carrageenan, which undergo sol–gel transitions in response to temperature changes. These polysaccharides form robust gels upon cooling, enabling facile processing and molding while maintaining compatibility [28].

Additionally, hydrogen bonding and self-assembly contribute to hydrogel formation in several algal polymers, generating networks through reversible interactions that can respond dynamically to environmental cues. Collectively, these physical crosslinking strategies offer gentle and cell-friendly routes to hydrogel fabrication, supporting their expanding use in injectable therapeutics, regenerative scaffolds, and controlled-release systems [29].

### 4.2. Chemical Crosslinking

**Figure 7:**
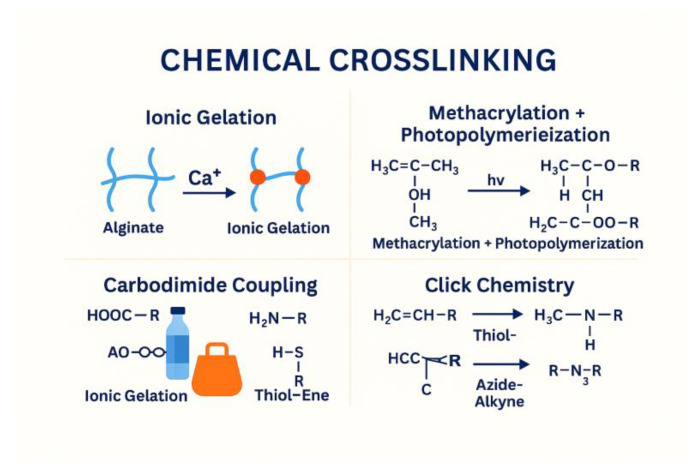
Chemical Crosslinking Mechanisms.

Chemical crosslinking strategies provide an alternative route to hydrogel fabrication, offering enhanced mechanical strength, structural integrity, and long-term stability compared with physically crosslinked systems [30]. These methods rely on the formation of covalent bonds within or between polymer chains, producing networks that are more resistant to degradation and mechanical deformation, qualities that are particularly valuable for load-bearing tissue scaffolds, long-term implants, and controlled-release platforms [31]. One widely used approach involves carbodiimide-mediated coupling, in which reagents such as EDC facilitate the formation of stable amide bonds between carboxyl and amine groups present in algal polysaccharides. This reaction proceeds under relatively mild conditions and enables precise tuning of crosslink density (Figure 7) [4].

Another important strategy is methacrylation followed by photopolymerization, where polysaccharides are functionalized with methacrylate groups and subsequently crosslinked through light-activated free-radical polymerization. This technique allows rapid gelation, spatial patterning, and compatibility with 3D bioprinting, making it highly versatile for advanced biomedical constructs [32]. Additionally, click chemistry reactions, including thiol–ene and azide–alkyne cycloadditions, offer highly efficient, selective, and biorthogonal crosslinking routes. These reactions proceed quickly and with minimal byproducts, enabling the fabrication of hydrogels with well-defined architecture and tailored mechanical properties. Collectively, chemical crosslinking methods expand the functional landscape of marine-derived hydrogels, providing robust and durable materials suited for demanding tissue engineering and therapeutic applications [33].

### 4.3. Composite and Hybrid Hydrogels

Composite and hybrid hydrogels represent an important advancement in the design of marine-derived biomaterials, allowing the intrinsic properties of phycocolloids to be enhanced or complemented through combination with other natural polymers or functional nanomaterials. By blending algal polysaccharides with biopolymers such as gelatin, collagen, or hyaluronic acid, it becomes possible to fine-tune key characteristics including bioactivity, mechanical stiffness, viscoelasticity, and degradation kinetics [7]. These hybrid systems often exhibit improved cell adhesion and biological signaling compared with phycocolloids alone, owing to the presence of extracellular-matrix-mimetic components that support tissue integration and regeneration [34].

In addition to biopolymer blends, the incorporation of nanomaterials, such as nanocellulose, hydroxyapatite, or graphene-based derivatives, further expands the functional landscape of these hydrogels [35]. Nanocellulose can reinforce the polymer network and enhance shear resistance, while hydroxyapatite contributes osteoconductive properties valuable for bone tissue engineering [36]. Graphene derivatives introduce electrical conductivity and mechanical reinforcement, enabling applications in neural, cardiac, or electroactive tissue scaffolds. Through these combinations, composite hydrogels achieve a level of tunability and multifunctionality that surpasses single-component systems, positioning them as versatile platforms for advanced biomedical therapeutics and regenerative medicine [37].

## 5. Applications

### 5.1. Skin and Wound Healing

Phycocolloid-based hydrogels have emerged as highly effective biomaterials for skin repair and wound management due to their intrinsic characteristics, moisture-retention capacity, and ability to form protective, conformable matrices over damaged tissue [38]. When applied to wounds, these hydrogels help maintain a moist microenvironment, one of the key determinants of accelerated healing, by preventing desiccation and supporting optimal cellular migration [39]. Many marine-derived hydrogels also exhibit inherent antimicrobial properties, either through their sulfated polysaccharide structures or through their ability to serve as carriers for antimicrobial agents. This contributes to reduced infection risk, a critical factor in chronic or complex wounds [40].

In addition to providing physical protection, phycocolloid hydrogels support enhanced re-epithelialization by facilitating keratinocyte proliferation and migration across the wound bed [41]. Their porous, hydrated networks can also be engineered to deliver bioactive molecules in a controlled manner, including growth factors, cytokines, and small-molecule therapeutics that modulate inflammation and promote tissue regeneration. This controlled-release capability positions marine-derived hydrogels as multifunctional dressings capable of both structural support and targeted therapeutic action [24].

**Figure 8:**
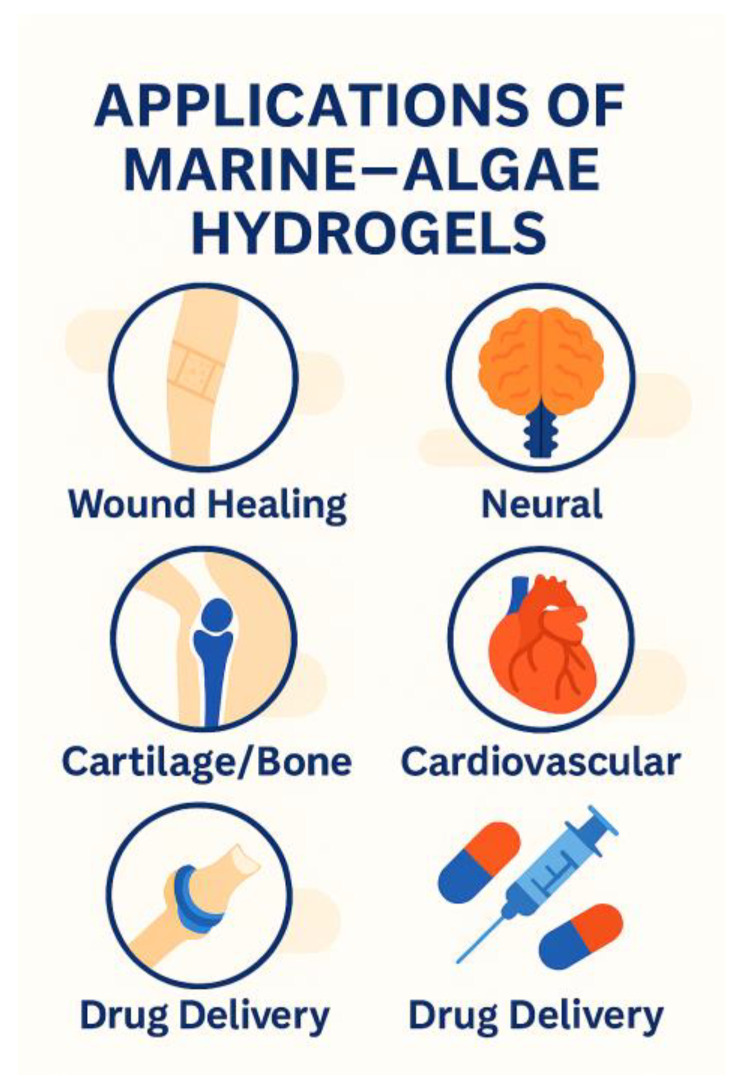
Biomedical applications of marine-algae-derived hydrogels, including wound healing, cartilage and bone regeneration, neural and cardiovascular repair, and drug delivery.

Among the various marine polymers, ulvan from green algae and extracellular polysaccharides (EPS) from Cyanophyceae have shown particular promise. Their highly sulfated, bioactive structures confer antioxidant, immunomodulatory, and antimicrobial activities that directly contribute to wound healing [42]. Ulvan-based hydrogels, for example, can modulate macrophage behavior and enhance collagen deposition, while cyanobacterial EPS exhibit strong free-radical scavenging and moisture-retention properties. Together, these features make phycocolloid hydrogels versatile platforms for next-generation wound dressings, combining structural functionality with intrinsic biological activity to support rapid and effective skin regeneration (Figure 8) [43].

### 5.2. Cartilage and Bone Regeneration

Marine-derived hydrogels have gained considerable attention in cartilage and bone tissue engineering due to their ability to mimic key features of the native extracellular matrix. Among these, alginate- and agarose-based hydrogels are particularly well established for cartilage regeneration. Their gentle gelation mechanisms and highly hydrated networks support chondrocyte viability, preserve the rounded cell morphology essential for maintaining the chondrogenic phenotype, and facilitate the deposition of cartilage-specific extracellular matrix components such as type II collagen and glycosaminoglycans. These hydrogels also provide a mechanically stable yet permissive environment that can be tailored to match the viscoelastic properties of native cartilage, making them suitable for both in vitro chondrogenesis and in vivo cartilage repair strategies [44].

For bone regeneration, composite hydrogels incorporating bioactive components have shown significant promise. The integration of hydroxyapatite nanoparticles, for example, enhances the osteoconductive properties of phycocolloid matrices, promoting mineral deposition and supporting the differentiation of mesenchymal stem cells toward osteogenic lineages [45]. Similarly, the incorporation of bioactive peptides, including RGD motifs or osteo-inductive sequences, improves cell adhesion, proliferation, and lineage-specific signaling, thereby strengthening the regenerative potential of these materials. These hybrid systems combine the favorable handling and injectability of algal hydrogels with the mechanical reinforcement and biological cues required for robust bone tissue formation [46].

These advances highlight the versatility of marine-derived hydrogels as platforms for musculoskeletal regeneration. By supporting chondrocyte function, guiding osteogenic differentiation, and enabling the incorporation of bioactive fillers, phycocolloid-based hydrogels offer a promising foundation for next-generation therapies targeting cartilage defects, osteochondral interfaces, and bone repair [47].

### 5.3. Neural Tissue Engineering

Neural tissue engineering demands biomaterials that combine softness and electrical responsiveness, properties that can be effectively achieved using phycocolloid-based hydrogels [48]. When marine-derived polysaccharides are blended with conductive polymers such as polypyrrole, polyaniline, or PEDOT, the resulting hybrid hydrogels exhibit mechanical characteristics similar to native neural tissue while providing the electrical conductivity required to support neuronal communication. These soft, conductive matrices create a permissive environment for neuronal adhesion, enabling neurons to anchor, spread, and maintain functional phenotypes within a hydrated three-dimensional scaffold [49].

In addition to supporting cell attachment, these hydrogels facilitate axonal guidance, a critical requirement for repairing damaged neural pathways. The combination of aligned polymer networks, stiffness, and electroconductive domains helps direct neurite extension and promotes the formation of organized neural networks. Such structural cues are essential for regenerating peripheral nerves and for developing in vitro neural models that recapitulate physiological architecture [50].

Conductive phycocolloid-based hydrogels also hold significant potential for electrical stimulation therapies, which are increasingly used to enhance neuronal survival, synaptic activity, and functional recovery following injury. The incorporation of conductive polymers allows these hydrogels to transmit low-intensity electrical signals, enabling controlled stimulation protocols that can modulate neural behavior and accelerate regeneration. These features position marine-derived conductive hydrogels as promising platforms for neural repair, neuro-prosthetic interfaces, and advanced bioelectronic therapies [51].

### 5.4. Vascular and Cardiac Applications

Marine-derived hydrogels are increasingly explored for vascular and cardiac tissue engineering due to their structural similarity to native extracellular matrix components and their ability to support cell survival, organization, and function. In particular, sulfated polysaccharides from marine algae closely mimic the biochemical behavior of glycosaminoglycans, enabling them to interact with growth factors, cytokines, and adhesion molecules that regulate vascular development and cardiac repair [52]. These materials can promote angiogenesis by stabilizing pro-angiogenic factors such as VEGF and FGF, enhancing endothelial cell migration and tubule formation. Their soft, hydrated networks also provide a supportive environment for cardiomyocyte function, helping maintain contractility and electrical coupling in engineered cardiac tissues. When combined with bioactive peptides, conductive fillers, or aligned scaffolding architectures, phycocolloid-based hydrogels can further enhance vascular integration and myocardial regeneration, positioning them as promising candidates for cardiac patches, injectable therapeutics, and vascular graft coatings [53].

## 6. Hydrogels for Drug Delivery and Therapeutics

Phycocolloid-based hydrogels offer a versatile platform for drug delivery due to their porosity and ability to form stable, hydrated networks capable of encapsulating a wide range of therapeutic agents [54]. Their polymeric architecture enables sustained and controlled release, allowing drugs to diffuse gradually through the hydrogel matrix or be released in response to environmental triggers such as pH, ionic strength, or temperature. This controlled-release behavior is particularly valuable for chronic conditions requiring long-term dosing or for localized therapies where maintaining therapeutic concentrations at the target site is essential [55].

These hydrogels also provide protection for sensitive therapeutics, including proteins, peptides, nucleic acids, and probiotics, shielding them from enzymatic degradation or harsh physiological conditions [56]. Their gentle gelation mechanisms, especially in alginate, carrageenan, and ulvan systems, allow encapsulation without compromising bioactivity. Furthermore, the inherent bio-adhesive properties of many marine polysaccharides enable targeted delivery to inflamed or diseased tissues, where interactions with mucosal surfaces or extracellular matrix components enhance retention and therapeutic efficacy [57].

Because of their mucoadhesive behavior, injectability, and adjustable pore structure, phycocolloid hydrogels are well suited for multiple administration routes, including oral, transdermal, intranasal, ocular, and parenteral delivery [54]. Their capacity to incorporate nanoparticles, liposomes, or bioactive ligands further expands their utility, enabling multifunctional delivery systems that combine controlled release with imaging, targeting, or immunomodulatory capabilities. Collectively, these features position marine-derived hydrogels as powerful tools for next-generation therapeutic delivery platforms [40].

## 7. Three-Dimensional Bioprinting and Advanced Bio-Fabrication

Marine-derived hydrogels have become increasingly prominent in the field of 3D bioprinting, where their rheological behavior and rapid gelation make them highly suitable as bioinks [58]. Many phycocolloids exhibit shear-thinning properties, allowing them to flow smoothly through printing nozzles under applied pressure while rapidly recovering viscosity upon deposition. This characteristic is essential for maintaining structural fidelity during layer-by-layer fabrication [59]. Additionally, the rapid gelation mechanisms of alginate, carrageenan, and related polysaccharides, whether through ionic crosslinking, thermal transitions, or self-assembly, enable immediate stabilization of printed constructs. Their cytocompatibility further supports the encapsulation and survival of mammalian cells during and after printing, making these materials attractive for fabricating living tissues [60,61].

Among marine polysaccharides, alginate remains the most widely used bioink, largely due to its predictable ionic crosslinking, mild processing conditions, and extensive track record in cell-laden printing. However, next-generation phycocolloids are gaining momentum [62]. Ulvan, with its sulfated and bioactive structure, offers improved cell–matrix interactions and immunomodulatory potential [20]. Carrageenan provides thermo-reversible gelation and mechanical properties, while engineered cyanobacterial EPS introduce customizable biochemical functionalities and enhanced printability. These emerging materials expand the design space for bioinks, enabling more sophisticated and biologically relevant constructions [63].

Current developments in the field point toward increasingly complex fabrication strategies. Multi-material printing allows the integration of distinct bioinks within a single construct, enabling spatial patterning of biochemical cues, mechanical gradients, or multiple cell types [64]. Gradient scaffolds are being developed to mimic the heterogeneous architecture of native tissues, such as osteochondral interfaces or vascularized muscle. Looking ahead, 4D bioprinting, which incorporates stimuli-responsive phycocolloids capable of dynamic shape change or functional transformation, represents a major frontier. These smart materials respond to environmental triggers such as pH, temperature, or ionic strength, enabling printed constructions to evolve over time in ways that more closely emulate natural developmental or healing processes [65].

These advances position marine-derived hydrogels as foundational materials for the next generation of bio-fabrication technologies, offering a combination of printability, biological functionality, and adaptability that is essential for engineering complex, living tissues [1].

## 8. Challenges and Future Perspectives

Despite the rapid expansion of fabrication strategies, a major limitation in the field remains the absence of standardized protocols for the physicochemical and biological characterization of marine-derived hydrogels. Variability in extraction methods, polymer purity, crosslinking conditions, and analytical techniques leads to inconsistencies that make it difficult to compare results across research groups. Parameters such as mechanical strength, degradation kinetics, swelling behavior, and bioactivity are often measured using non-uniform methodologies, hindering reproducibility and slowing progress toward regulatory approval and clinical translation. Establishing harmonized characterization frameworks will therefore be essential for enabling meaningful cross-study comparisons and accelerating the development of reliable, clinically relevant hydrogel systems.

One of the most persistent issues is the batch-to-batch variability inherent to natural extracts [40]. The biochemical composition of phycocolloids can fluctuate depending on species, season, geographic origin, and extraction conditions, leading to inconsistencies in gelation behavior, mechanical properties, and bioactivity. This variability is compounded by the complex purification requirements needed to remove proteins, pigments, salts, and other coextracted compounds that may interfere with hydrogel performance. Achieving reproducible, high-purity polysaccharide fractions remains a major technical hurdle [66].

Another limitation is the relatively low mechanical strength of many marine-derived hydrogels, which restricts their use in load-bearing tissues such as bone, cartilage, or tendons [67]. Although composite and chemically crosslinked systems have improved mechanical performance, further innovation is needed to match the structural demands of these tissues without compromising degradability [68]. Additionally, the field lacks standardized characterization protocols, making it difficult to compare results across studies or establish clear structure–function relationships. Harmonized guidelines for rheology, degradation, purity assessment, and biological testing would greatly enhance reproducibility and accelerate regulatory acceptance [69].

Looking ahead, several promising research directions may help overcome these challenges. Genetic engineering of algae and Cyanophyceae offers a powerful route to producing tailored polysaccharides with controlled sulfation patterns, molecular weights, and functional groups, enabling unprecedented precision in hydrogel design [70]. Advances in hybrid hydrogel systems, combining phycocolloids with bioactive peptides, nanomaterials, or synthetic polymers, are expected to yield materials with enhanced mechanical robustness, biological signaling, and stimuli-responsive behavior. Progress toward clinical translation will require deeper engagement with regulatory frameworks, including rigorous safety assessments and scalable manufacturing processes [7].

Finally, the future success of marine-derived hydrogels depends on sustainable large-scale production. Cultivation of macroalgae, microalgae, and engineered Cyanophyceae in controlled bioreactors or offshore farms could provide reliable, environmentally responsible sources of high-quality polysaccharides. Integrating sustainability with advanced biomaterial engineering will be essential for positioning phycocolloid-based hydrogels as viable, next-generation materials for regenerative medicine and therapeutic delivery [71].

## 9. Conclusions

Marine-derived hydrogels represent a rapidly evolving class of biomaterials with exceptional potential across tissue engineering, regenerative medicine, and therapeutic delivery. Their unique physicochemical properties, arising from the diverse polysaccharide structures of macroalgae, microalgae, and Cyanophyceae, enable the fabrication of hydrogels with mechanics, high tolerance, and intrinsic bioactivity. Advances in physical, chemical, and hybrid crosslinking strategies have expanded the functional landscape of these materials, allowing the creation of scaffolds that support cell viability, guide tissue regeneration, and respond dynamically to physiological cues. Applications now span skin repair, cartilage and bone regeneration, neural and cardiovascular engineering, and controlled drug delivery, demonstrating the versatility and translational promise of phycocolloid-based systems [52].

At the same time, the integration of marine hydrogels into emerging technologies such as 3D bioprinting and advanced bio-fabrication underscores their relevance for next-generation biomedical constructs. Their shear-thinning behavior, rapid gelation, and cytocompatibility make them ideal candidates for cell-laden bioinks, while innovations in multi-material printing, gradient scaffolds, and stimuli-responsive “4D” architectures continue to push the boundaries of what these materials can achieve [72].

Despite these advances, key challenges remain, including variability in natural extracts, purification complexity, limited mechanical strength for load-bearing applications, and the need for standardized characterization protocols. Addressing these limitations will require coordinated efforts in biotechnology, materials science, and regulatory science. Future progress is likely to be driven by genetic engineering of algae and cyanobacteria to produce tailored polysaccharides, the development of multifunctional hybrid hydrogels, and the establishment of sustainable large-scale production systems capable of delivering consistent, high-quality biomaterials [73].

Overall, marine-derived hydrogels stand at the forefront of a new generation of sustainable, bioactive, and highly adaptable biomaterials. Their continued development promises to unlock innovative therapeutic strategies and contribute meaningfully to the future of regenerative medicine and biomedical engineering [74].

## Data Availability

No new data were created or analyzed in this study. Data sharing is not applicable to this article.

## Abbreviations

The following abbreviations are used in this manuscript:

3D	Three-Dimensional
4D	Four-Dimensional
EDC	1-Ethyl-3-(3-dimethylaminopropyl) carbodiimide hydrochloride
EPS	Extracellular Polysaccharides
FGF	Fibroblast Growth Factor
PEDOT	Poly(3,4-ethylenedioxythiophene)
VGEF	Vascular Endothelial Growth Factor
